# Association of cardiovascular risk profile with premature all-cause and cardiovascular mortality in US adults: findings from a national study

**DOI:** 10.1186/s12872-023-03672-3

**Published:** 2024-02-06

**Authors:** Ryan T. Nguyen, Vardhmaan Jain, Isaac Acquah, Safi U. Khan, Tarang Parekh, Mohamad Taha, Salim S. Virani, Michael J. Blaha, Khurram Nasir, Zulqarnain Javed

**Affiliations:** 1 Department of Medicine, Houston Methodist, Houston, TX US; 2 Department of Cardiovascular Medicine, Emory University School of Medicine, Atlanta, GA US; 3 Methodist DeBakey Heart and Vascular Center, Houston, TX US; 4 Department of Cardiovascular Medicine, Houston Methodist DeBakey Heart and Vascular Center, Houston, TX US; 5 Cardiovascular Prevention and Wellness, Department of Cardiovascular Medicine, Houston, US; 6 Center for Health Data Science and Analytics, Houston Methodist, Houston, TX US; 7 Department of Medicine, The Aga Khan University, Karachi, Pakistan; 8 Department of Cardiology, Texas Heart Institute, Baylor College of Medicine, Houston, TX US; 9 Department of Cardiology, Johns Hopkins University, Baltimore, MD US; 10 Center for Cardiovascular Computational Health and Precision Medicine, Houston Methodist, Houston, TX USA; 11 Houston Methodist Academic Institute, Houston, TX USA

**Keywords:** Cardiovascular risk profile, Cardiovascular mortality, Coronary Heart Disease

## Abstract

**Objective:**

To assess the association between cardiovascular risk factor (CRF) profile and premature all-cause and cardiovascular disease (CVD) mortality among US adults (age < 65).

**Methods:**

This study used data from the National Health Interview Survey from 2006 to 2014, linked to the National Death Index for non-elderly adults aged < 65 years. A composite CRF score (range = 0–6) was calculated, based on the presence or absence of six established cardiovascular risk factors: hypertension, diabetes, hypercholesterolemia, smoking, obesity, and insufficient physical activity. CRF profile was defined as “Poor” (≥ 3 risk factors), “Average” (1–2), or “Optimal” (0 risk factors). Age-adjusted mortality rates (AAMR) were reported across CRF profile categories, separately for all-cause and CVD mortality. Cox proportional hazard models were used to evaluate the association between CRF profile and all-cause and CVD mortality.

**Results:**

Among 195,901 non-elderly individuals (mean age: 40.4 ± 13.0, 50% females and 70% Non-Hispanic (NH) White adults), 24.8% had optimal, 58.9% average, and 16.2% poor CRF profiles, respectively. Participants with poor CRF profile were more likely to be NH Black, have lower educational attainment and lower income compared to those with optimal CRF profile. All-cause and CVD mortality rates were three to four fold higher in individuals with poor CRF profile, compared to their optimal profile counterparts. Adults with poor CRF profile experienced 3.5-fold (aHR: 3.48 [95% CI: 2.96, 4.10]) and 5-fold (aHR: 4.76 [3.44, 6.60]) higher risk of all-cause and CVD mortality, respectively, compared to those with optimal profile. These results were consistent across age, sex, and race/ethnicity subgroups.

**Conclusions:**

In this population-based study, non-elderly adults with poor CRF profile had a three to five-fold higher risk of all-cause and CVD mortality, compared to those with optimal CRF profile. Targeted prevention efforts to achieve optimal cardiovascular risk profile are imperative to reduce the persistent burden of premature all-cause and CVD mortality in the US.

## Introduction

Cardiovascular disease (CVD) is the leading cause of mortality globally [1–3, 13]. Despite the decline in all-cause and CVD mortality over the past century, attributable in part to evidence-based prevention efforts and guideline-directed medical care, the gains in life expectancy have halted in recent years, as evidence by a slowing rate of decline in mortality among non-elderly individuals (age < 65) since 2011 [4–7]. These patterns may be explained by the rise in prevalence of modifiable risk factors, including diabetes, obesity, and hypertension among non-elderly adults [8, 9].

There is increasing evidence to suggest a mortality benefit of cardiovascular risk factor control; however, current knowledge is predominantly based on the general US population, without due attention to all-cause and CVD mortality in the non-elderly (premature mortality) [10]. Additionally, most prior studies have reported the effect(s) of individual CVD risk factors; the association between cardiovascular risk factor (CRF) control and premature all-cause and CVD mortality has not been studied using a composite measure of CRF profile. Despite the established concept of metabolic syndrome [11], relatively few studies have assessed the cumulative effects of a composite cardiovascular risk factor index – including diabetes, hypertension, dyslipidemia, obesity, physical inactivity and smoking – on premature mortality on a population scale in the US [12]. Finally, little is known about potential variation in the aforementioned association by age, sex, and racial/ethnic subgroups. To our knowledge, these associations have not been assessed in a nationally representative sample of non-elderly US adults.

A deeper understanding of the composite effect of multiple, often interlinked risk factors on all-cause and CVD mortality is needed to identify clinically vulnerable population subgroups and inform the design of primary prevention efforts to reduce the burden of premature mortality in the US. In this population-based study, we sought to evaluate the association between a composite measure of CRF control and risk of premature all-cause and CVD mortality. We also assessed potential variation in the CRF-mortality association by key sociodemographic subgroups.

## Methods

### Data sharing statement

All data used in this study are publicly available [13, 14].

### Study design, setting, and population

We used data for non-elderly adults (aged 18–64 years) from the 2006–2014 National Health Interview Survey (NHIS), linked to the National Death Index (NDI) for mortality ascertainment [11]. The NHIS is an annually updated, cross-sectional database that uses complex, multistage probability sampling, incorporating stratification, oversampling, and clustering to provide national level estimates of the US non-institutionalized population. The NHIS collects standardized information on race, ethnicity, sex, primary language, disability status, and is an important source of information on health disparities and healthcare resource utilization in the US.

The survey consists of four core questionnaires: the household composition, the family core, the sample adult core, and the sample child core. The household section collects basic demographic and relationship information about all persons in the household. The family core collects data on basic sociodemographic characteristics, indicators of health status, activity limitations, injuries, insurance coverage, and access to and utilization of health services. From each family, one sample child and one sample adult are randomly selected to gather further information. In this study, we used the sample adult file with supplementation of variables from other cores. Because of the de-identified nature and public availability of the NHIS, this study was considered exempt from the Institutional Review Board of Houston Methodist Hospital.

*Inclusion/exclusion criteria*: We included NHIS participants aged 18 years and over. Participants with unavailable information on death status or insufficient identifying data (< 2%) were not eligible for linkage to the NHIS and were therefore excluded from the study population. We restricted the final sample to the following racial/ethnic subgroups: non-Hispanic White, non-Hispanic Black and Hispanic; individuals from other racial/ethnic backgrounds (Asian/others) were excluded due to small sample size (≈ 1% of the total population).

### Study variables

#### CRF profile

Individual traditional risk factors (hypertension, diabetes mellitus, high cholesterol, smoking, obesity, and insufficient physical activity) were ascertained by self-report based on the following survey questions: (1) hypertension: “Have you ever been told by a doctor or other health professional that you have hypertension, also called high blood pressure?”; (2) diabetes: “Has a doctor or other health professional ever told you that you had diabetes or high blood sugar?”; (3) high cholesterol: “Have you ever been told by a doctor or other health professional that you had high cholesterol?”; (4) Smoking was ascertained as an individual being either former or current smoker; (5) obesity (defined as a body mass index ≥ 30 kg/m^2^) was calculated based on self-reported height and weight; (6) Insufficient physical activity was defined as not meeting the current physical activity guidelines (i.e. not participating in moderate-intensity aerobic physical activity for > 150 min per week, or vigorous-intensity aerobic physical activity for > 75 min per week, or a total combination of ≥ 150 min per week of moderate/vigorous-intensity aerobic physical activity [15].

Each of the six components was scored 0/1 depending on the presence or absence of the risk factor. A composite CRF score was created by aggregating individual risk factors, with the final score ranging from 0 to 6. Finally, participants’ CRF profile was defined as: “poor” (≥ 3 risk factors), “average” (1–2), or “optimal” (0).

#### All-cause and CVD mortality

The primary outcomes of this study were all-cause and CVD mortality. Mortality was ascertained using participants’ death certificate records in the NDI. A participant was defined as dead if identified as ‘deceased’ in the NDI during the study follow-up period. Cause of death was determined using the International Classification of Diseases, 9th and 10th Revisions. All-cause mortality was defined as death due to any underlying cause. CVD mortality was defined as death due to ‘diseases of the heart’ (ICD = I00-I09, I11, I13, I20-I51) or ‘cerebrovascular diseases’ (ICD = I60-I69).

#### Sociodemographic characteristics

Race and Ethnicity were defined by self-report and were categorized as Non-Hispanic (NH) White, non-Hispanic (NH) Black, and Hispanic. Age was categorized into three groups: 18–44 years, 45–54 years, and 55–64 years old. Sex was divided by Men and Women. Covariates included family income, defined as a percentage of the federal poverty limit and was categorized as high (> 400%), middle (200% to < 400%), or low (< 200%) income status [16, 17], insurance status (insured or uninsured), educational attainment (< High School, High School/GED, Some college, > College level of education), and geographic region (Northeast, Midwest, South, or West). Comorbidities were ascertained (also self-reported), and included chronic obstructive pulmonary disease, asthma, gastrointestinal ulcer, arthritis (including arthritis, gout, fibromyalgia, rheumatoid arthritis and systemic lupus erythematosus), cancer (any), any kind of liver condition or “weak/failing” kidneys. Number of prevalent comorbidities were summed for each individual and stratified into 0, 1 and ≥ 2.

### Statistical analysis

We used survey-specific descriptive statistics to obtain weighted national estimates of study participants based on the CRF profile (Poor, Average, or Optimal) across sociodemographic characteristics. Rao-Scott Chi squared tests were used to compare characteristics across categories of CRF profile. Continuous variables were described as mean with standard deviations and categorical variables were described as proportions. Poisson regression was used to generate age-adjusted mortality rates (AAMR, per 100,000 person-years (PYs)) with 95% confidence intervals (CI) for all-cause and CVD mortality. AAMR were reported for each CRF profile category in the total population, and by age, sex and race/ethnicity. Kaplan Meier curves were generated to report the survival probability across CRF profile, separately for all-cause and CVD mortality.

Cox proportional hazards regression models were used to generate unadjusted and adjusted (age, sex, education, income, insurance status, and race/ethnicity) hazard ratios (HRs) and 95% CI for all-cause and CVD mortality. Stratified analyses by age, sex, race/ethnicity, education, income, and insurance status were conducted by Stata 17, accounting for survey design effects.

We also performed additional sensitivity analysis adjusting for additional SDoH factors including insurance status, region, delayed/forgone care due to cost and food insecurity, and comorbidities including ASCVD, cancer, and sleep trouble. Delayed care was defined as care that was delayed and/or forgone care due to cost. Food insecurity was defined as a composite of a 10 item food insecurity questionnaire derived from the United States Department of Agriculture (USDA) screening tool, defined as binary (food insecurity = yes versus no) variable. The definition of cancer included any cancer type. Trouble sleeping was defined as ≥ 1 night with difficulty falling asleep in the past week.

## Results

### Descriptive characteristics

Study population comprised 195,901 participants, representing 183,726,661 non-elderly US adults (Table 1). Over half of the study population were young (18–44 years, 58.6%) adults, 51% female, 70% NH White, 13.3% NH Black, and 16.7% Hispanic adults. In the total population, 24.8% of participants had optimal, 58.9% average, and 16.2% had poor CRF profile. Participants with poor CRF profile were more likely to be NH Black (18.0% vs. 9.3%), have lower levels of education (19.0% vs. 6.8% < high school and 34.3% vs. 16.6% Highschool or GED), had low family income (40.0% vs. 22.1%), compared to those with an optimal CRF profile. Older individuals (55–64 years) were more likely to have a poor CRF profile (35.6% vs. 11.4%) compared to optimal CRF profile. There were no significant differences of CRF profile between males and females. Individuals with poor CRF profile were five-fold as likely to report two or more comorbidities, compared to those with optimal CRF profile.

**Table 1:** Descriptive Characteristics by CRF categories among Non-Elderly Adults, from the National Health Interview Survey 2006-14

	Overall	Optimal	Average	Poor	*p* value
**Sample (N)**	195,901	46,701	114,961	34,239	
**Weighted sample, (weighted %)**	183,726,611 (100.0)	45,549,963 (24.8)	108,375,333 (58.9)	29,801,315(16.2)	
**Mean age (SD)**	40.4 (13.0)	36.5 (12.4)	39.9 (12.7)	48.2 (11.6)	
**Age Categories**					< 0.001
18–44	113,320 (58.6)	33,121 (70.1)	69,298 (60.7)	10,901 (33.2)	
45–54	43,425 (22.8)	8148 (18.6)	25,072 (22.3)	10,205 (31.2)	
55–64	39,156 (18.6)	5432 (11.4)	20,591 (17)	13,133 (35.6)	
**Sex, n (weighted %)**					0.845
Men	89,079 (49.1)	21,799 (49.2)	52,140 (49.1)	15,140 (49)	
Women	106,822 (50.9)	24,902 (50.8)	62,821 (50.9)	19,099 (51)	
**Race/Ethnicity, n (weighted %)**					< 0.001
Non-Hispanic White adults	112,084 (70)	29,630 (75.9)	62,973 (68)	19,481 (68.7)	
Non-Hispanic Black adults	31,222 (13.3)	4808 (9.3)	18,503 (13.7)	7911 (18)	
Hispanic adults	38,552 (16.7)	8068 (14.8)	25,025 (18.4)	5459 (13.3)	
**Education, n (weighted %)**					< 0.001
< High School	28,732 (13.3)	3237 (6.8)	18,402 (14.4)	7093 (19)	
High School or GED	49,468 (26.2)	7463 (16.6)	30,838 (28)	11,167 (34.3)	
Some College	40,161 (20.8)	9964 (21.8)	23,329 (20.6)	6868 (20)	
> College	76,729 (39.7)	25,936 (54.8)	41,841 (37)	8952 (26.7)	
**Family income, n (weighted %)**					< 0.01
High-income	60,948 (39.8)	19,665 (51.9)	33,477 (37.4)	7806 (30.1)	
Middle-income	49,681 (29)	11,275 (26)	29,820 (30.1)	8586 (29.9)	
Low-income	66,723 (31.2)	11,794 (22.1)	40,205 (32.6)	14,724 (40)	
**Insurance status, n (weighted %)**					< 0.001
Insured	145,435 (79.4)	37,333 (85)	82,358 (76.7)	25,744 (80.3)	
Uninsured	41,819 (20.6)	7472 (15)	27,780 (23.3)	6567 (19.7)	
**Region, n (weighted %)**					< 0.001
Northeast	31,260 (17.3)	7507 (17.5)	18,673 (17.6)	5080 (15.8)	
Midwest	42,408 (23.5)	9904 (23)	24,800 (23.4)	7704 (24.5)	
South	71,899 (36.5)	14,948 (32)	42,427 (36.8)	14,524 (42.3)	
West	50,334 (22.8)	14,342 (27.5)	29,061 (22.3)	6931 (17.4)	
**Cardiovascular Risk Factors, n (weighted %)**					< 0.001
Diabetes mellitus	13,812 (6.5)	-	2844 (2.5)	10,968 (31.2)	
Hypertension	45,990 (22.4)	-	19,903 (17.2)	26,087 (75.5)	
High cholesterol	15,476 (19.7)	-	5973 (14.3)	9503 (60.2)	
Smoker	42,284 (21.3)	-	27,190 (24)	15,094 (44.2)	
Obesity	60,789 (30.3)	-	34,894 (30.3)	25,895 (76.7)	
Insufficient physical activity	101,128 (50.7)	-	71,673 (62.2)	29,455 (86.3)	
**Comorbidities**					< 0.001
0	50,869 (66.8)	14,364 (77.7)	29,557 (69.4)	6948 (45.3)	
1	18,621 (23.8)	3520 (18.8)	9949 (23)	5152 (32.5)	
>=2	8085 (9.4)	691 (3.4)	3498 (7.6)	3896 (22.2)	

Note: Optimal CRF Profile = 0 CV risk factors; Average = 1–2 risk factors; Poor ≥ 3 risk factors

Comorbidities include Emphysema, COPD, Asthma, Ulcer, Cancer, Arthritis, Hepatitis, Liver disease, Kidney disease

Rao-Scott Chi squared tests were used to analyze the statistical data in this table

**Fig. 1:**
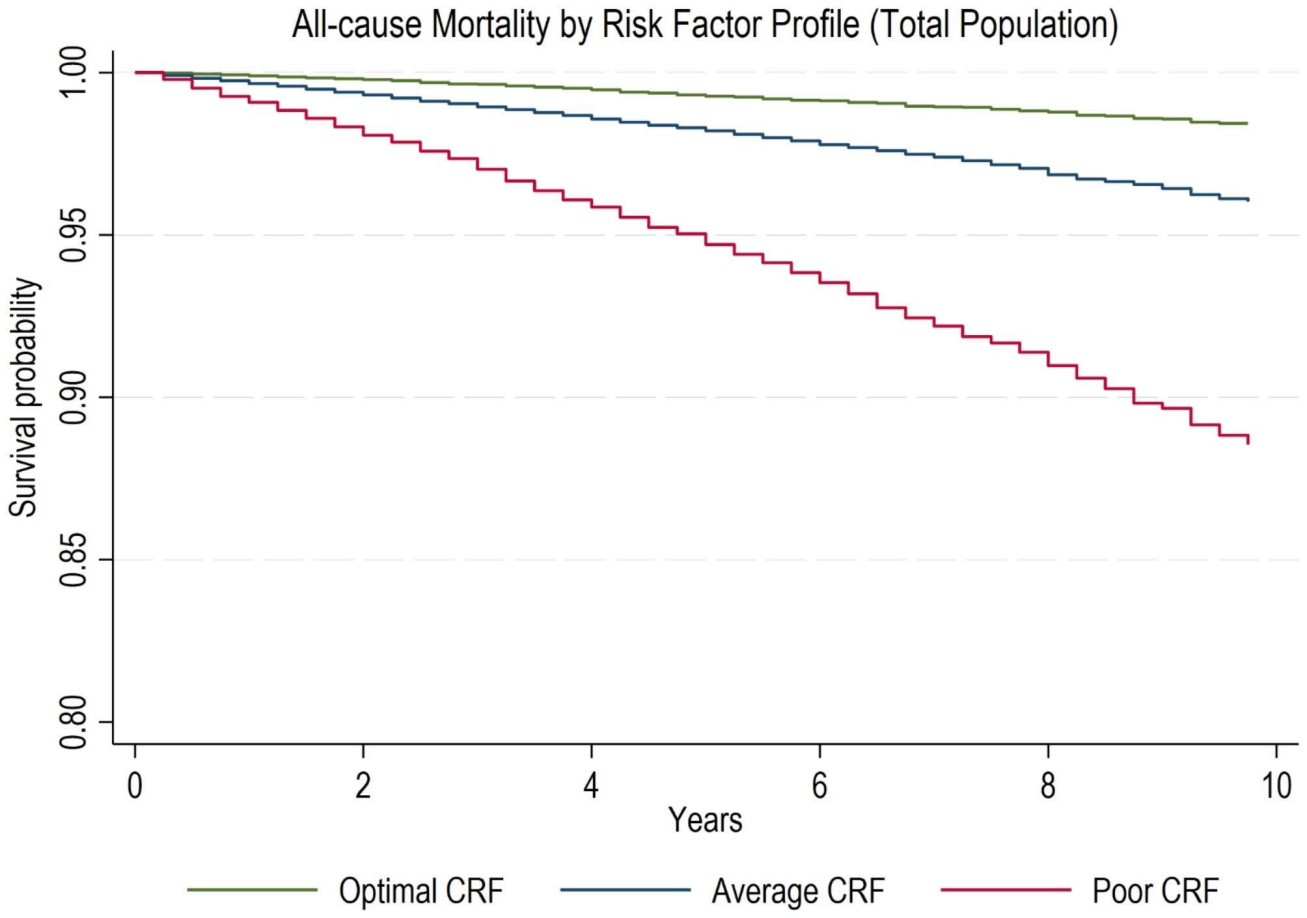
KM Curve for All-cause Mortality by Risk Factor Profile Note: Adjusted for sociodemographic factors including sex, race/ethnicity, education, SES, insurance status

**Fig. 2:**
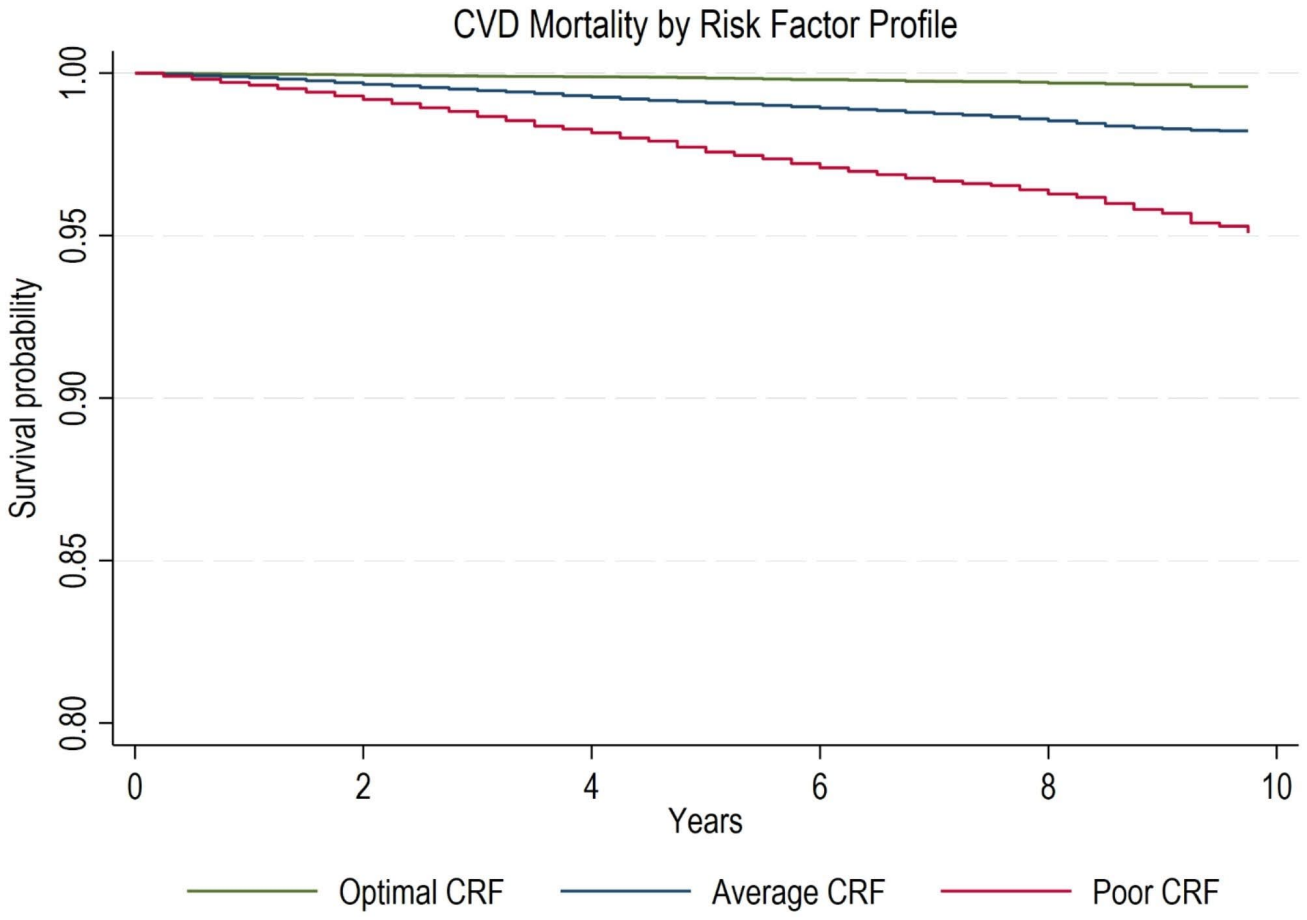
KM Curve for CVD mortality by Risk Factor Profile Note: Adjusted for sociodemographic factors including sex, race/ethnicity, education, SES, insurance status

Kaplan-Meier analysis revealed that the estimated survival probability for both all-cause and CVD mortality was highest for individuals with optimal CRF profile and lowest for those with poor profile (Figs. 1 and 2).

### AAMR

#### All-cause mortality

Table 2 presents all-cause mortality rates across CRF profile categories and sociodemographic subgroups. Participants with poor CRF profile had the highest AAMR, followed respectively by those with average and optimal CRF profiles (838.7 vs. 397.8 vs. 177.0); these trends were observed across age, sex, and race/ethnicity strata. For each CRF profile category, AAMR were higher for the middle aged, relative to younger adults (1800 vs. 1066 vs. 441.7 for poor, 857.8 vs. 520 vs. 206.1 for Average, and 319.4 vs. 205.3 vs. 110.2 for Optimal CRF profile) and men compared to women (976.7 vs. 708.6 for Poor, 480.3 vs. 317.2 for Average, and 226.9 vs. 131.1 for Optimal CRF profile). AAMR were higher for NH Black adults vs. NH White adults at optimal/average profile (217.9 vs. 139.3 for Optimal and 462.7 vs. 381.2 for Average). AAMR were lowest for the Hispanic population within the poor CRF profile (701.6). However, among the optimal and average CRF profiles, the Hispanic population exhibited the highest all-cause mortality at 336.8 and 519.7, respectively. AAMR increased with worsening CRF profile across all education and income strata. Additionally, AAMR were higher for those with lower education and/or income, at any given level of CRF profile. Individuals who had a poor CRF profile and were from the South had the highest AAMR (921.6). AAMR increased in a step wise fashion for increasing number of comorbidities across all CRF profiles.

**Table 2:** Age-adjusted mortality rates by sociodemographic subgroups, from the national health interview survey 2006–2014

	All-cause mortality (per 100,000 person-years)			CVD mortality (per 100,000 person-years)		
	Optimal	Average	Poor	Optimal	Average	Poor
**Mortality Rate**	177.0 (154.9–199.1)	397.8 (378.5–417.2)	838.7 (786.5–890.8)	74.0 (57.5–90.6)	197.7 (185.5–209.8)	328.6 (303.5–353.7)
**Age Categories**						
18–44	110.2 (90.2–130.1)	206.1 (187.9–224.3)	441.7 (368.6–514.9)	3.1 (0.6–5.7)	17.8 (12.9–22.8)	51.4 (30.6–72.1)
45–54	205.3 (157.4–253.1)	520 (476.4–563.6)	1066 (943.1–1190)	27.3 (11.1–43.5)	93 (70.6–115.5)	194.1 (145.8–242.5)
55–64	319.4 (237.2–401.6)	857.8 (792.2–923.3)	1800 (1658–1942)	54.1 (21.4–86.8)	153.5 (123–184)	346.3 (280.9–411.7)
**Sex, n (weighted %)**						
Men	226.9 (190.2–263.6)	480.3 (451–509.6)	976.7 (897.1–1056)	88.8 (64.3–113.3)	219.1 (200.7–237.6)	391.6 (350.5–432.8)
Women	131.1 (107.1–155.0)	317.2 (291.9–342.5)	708.6 (643.1–774)	57 (35.4–78.6)	178.8 (162.6–195)	280.7 (251.1–310.3)
**Race/Ethnicity, n (weighted %)**						
NH White adults	139.3 (114.1–164.6)	381.2 (356.2–406.2)	858.1 (793.3–922.9)	79.6 (59.5–99.8)	227.8 (211–244.6)	382.3 (346.8–417.9)
NH Black adults	217.9 (153.1–282.8)	462.7 (417.2–508.3)	828.7 (673.8–983.6)	85.3 (22.2–148.5)	161.6 (132.2–191.1)	264.2 (240.2–288.1)
Hispanic adults	336.8 (271.8–401.2)	519.7 (474.5–565.1)	701.6 (650.1–752.9)	48.6 (17.8–79.1)	105.1 (68.2–143.0)	180.8 (150.1–211.2)
**Education, n (weighted %)**						
< High School	397.7 (280.1–515.3)	604.2 (545.5–662.8)	1163 (1028–1299)	160.6 (69–252.3)	405.7 (359.9–451.5)	615.7 (542.8–688.7)
High School or GED	249.6 (182.8–316.3)	468.1 (424.7–511.6)	911.2 (821.7–1001)	95.6 (49.4–141.8)	224.1 (198.5–249.6)	321 (276.5–365.5)
Some College	148.2 (109.3–187.2)	330.9 (289.8–372.1)	635.2 (545.5–724.9)	68.1 (35.3–100.9)	139.7 (117.8–161.6)	274 (221.3–326.6)
> College	153.8 (127.8–179.7)	297.7 (270.2–325.1)	612.9 (521.3–704.6)	50.7 (32.0–69.4)	129 (112.5–145.5)	215.5 (180.2–250.8)
**Family income, n (weighted %)**						
High-income	145.8 (118.9–172.7)	276 (248.8–303.1)	530.1 (452.1–608.1)	32.8 (16.1–49.5)	112 (96.1–128)	181.8 (142.2–221.4)
Middle-income	163.3 (115.2–211.5)	395.5 (357–434.1)	752.5 (653.3–851.8)	57.9 (29.9–85.9)	208.5 (180.3–236.7)	314.5 (267.9–361.2)
Low-income	343.6 (258.4–428.8)	551.5 (508.5–594.5)	952.1 (867.2–1037)	135.5 (79.4–191.6)	244.9 (217.9–271.9)	417.7 (370.5–464.8)
**Insurance status, n (weighted %)**						
Insured	155.3 (132.1–178.6)	384.5 (362.5–406.6)	856.2 (794.2–918.1)	84.8 (64.2–105.3)	227.6 (213.1–242.1)	380.7 (350.3–411.2)
Uninsured	326.3 (253.2–399.5)	412 (367.5–456.5)	730.9 (623–838.8)	39.7 (12.8–66.6)	73.5 (53.5–93.4)	129.5 (88.2–170.7)
**Region, n (weighted %)**						
Northeast	160.6 (98.76–222.5)	370.7 (326.1–415.4)	665.1 (543.2–787)	103.6 (43.9–163.4)	215.3 (189.9–240.7)	288.5 (231.6–345.5)
Midwest	157.5 (113.2–201.8)	368.8 (329.3–408.2)	795.7 (696.6–894.9)	68.8 (40.6–97.1)	193.3 (164.1–222.5)	363.3 (305.5–421.1)
South	181.4 (143.7–219.0)	409.7 (374.8–444.7)	921.6 (839.4–1004)	62.6 (37.0–88.1)	198.1 (177.5–218.8)	351.1 (309.9–392.4)
West	197.9 (156.3–239.6)	434 (394.4–473.7)	841.1 (704.0–978.2)	77.6 (44.6–110.6)	187.8 (164–211.5)	274.6 (225.7–323.4)
**Cardiovascular Risk Factors, n (weighted %)**						
Diabetes mellitus		872.2 (693.1–1051)	1653 (1501–1805)		511.2 (404.8–617.5)	792.8 (709.3–876.2)
Hypertension		561.6 (509.4–613.7)	1235 (1156–1315)		390.6 (355.7–425.5)	668.9 (617.7–720.1)
High cholesterol		335.1 (193.1–477.2)	833.2 (692.2–974.2)		179 (91.6–266.4)	342 (270.7–413.3)
Smoker		588.4 (542.2–634.6)	1007 (920.3–1094)		158.1 (131–185.1)	262.4 (224.7–300.1)
Obesity		331.1 (295.5–366.7)	815.5 (752.1–878.9)		134.9 (107.7–162.1)	266 (239–293.1)
Insufficient physical activity		466.5 (438.7–494.3)	918.8 (860.5–977.1)		280.3 (260–300.6)	427.1 (393.8–460.4)
**Comorbidities**						
0	138.1 (82.42–193.8)	222.0 (184.5–259.4)	436.5 (289.5–583.6)	40.7 (7.1–74.4)	97.8 (70.0–125.5)	110.2 (69.1–151.3)
1	175.8 (68.8–282.7)	525.1 (398.1–652.0)	739.6 (540.7–938.5)	25.8 (1.3–61.9)	158.2 (114.6–201.8)	286 (200.9–371.1)
>=2	539.7 (519.7–558.7)	1185 (1074.3–1295.0)	1405 (1123.1–1687.0)	64.3 (9.2–157.7)	310.7 (208.2–413.1)	510.1 (375–645.1)

#### CVD mortality

*A*ge-Adjusted mortality rates (AAMR) for cardiovascular disease (CVD) mortality are presented in Table 2. In terms of CVD mortality, we noted similar trends with higher age-adjusted CVD mortality rates per 100,000 person-years in participants with poor CRF profile, followed by those with average and optimal CRF profile (328.6 vs. 197.7 vs. 74.0). These trends were observed across age, sex, and race/ethnicity strata. Among participants with a poor CRF profile, individuals aged 55–64 had higher CVD mortality rates compared to individuals aged 45–54 and 18–44 years (346.3 vs. 194.1 vs. 51.4). Similar trends were noted for individuals with an optimal or average CRF profile where advancing age carried higher CVD mortality rates. Men had higher CVD mortality rates compared to women across all CRF profile groups (391.6 vs. 280.7 for Poor, 219.1 vs. 178.8 for Average, and 88.8 vs. 57.0 for Optimal CRF profiles).

AAMR were higher for NH White adults vs. NH Black adults at Average/Poor profile (227.8 vs. 161.6 for Average and 382.3 vs. 264.2 for Poor) and relatively lower at optimal CRF profile (79.6 vs. 85.3). AAMR were lowest for the Hispanic population in the average and poor CRF profiles compared to NH Black and White adults. Similar to All-cause AAMR, there were higher CVD AAMR with worsening CRF profile across all education and income strata. Individuals who had a poor CRF profile and were from the Midwest had the highest AAMR (363.3) with those from the South having the second highest AAMR (351.1). Similar to the findings in all-cause mortality, AAMR increased in a stepwise fashion for increasing co-morbidities across all CRF profiles.

### Multivariable regression

#### Association between cardiovascular risk factor (CRF) profile and all-cause mortality

**Fig. 3:**
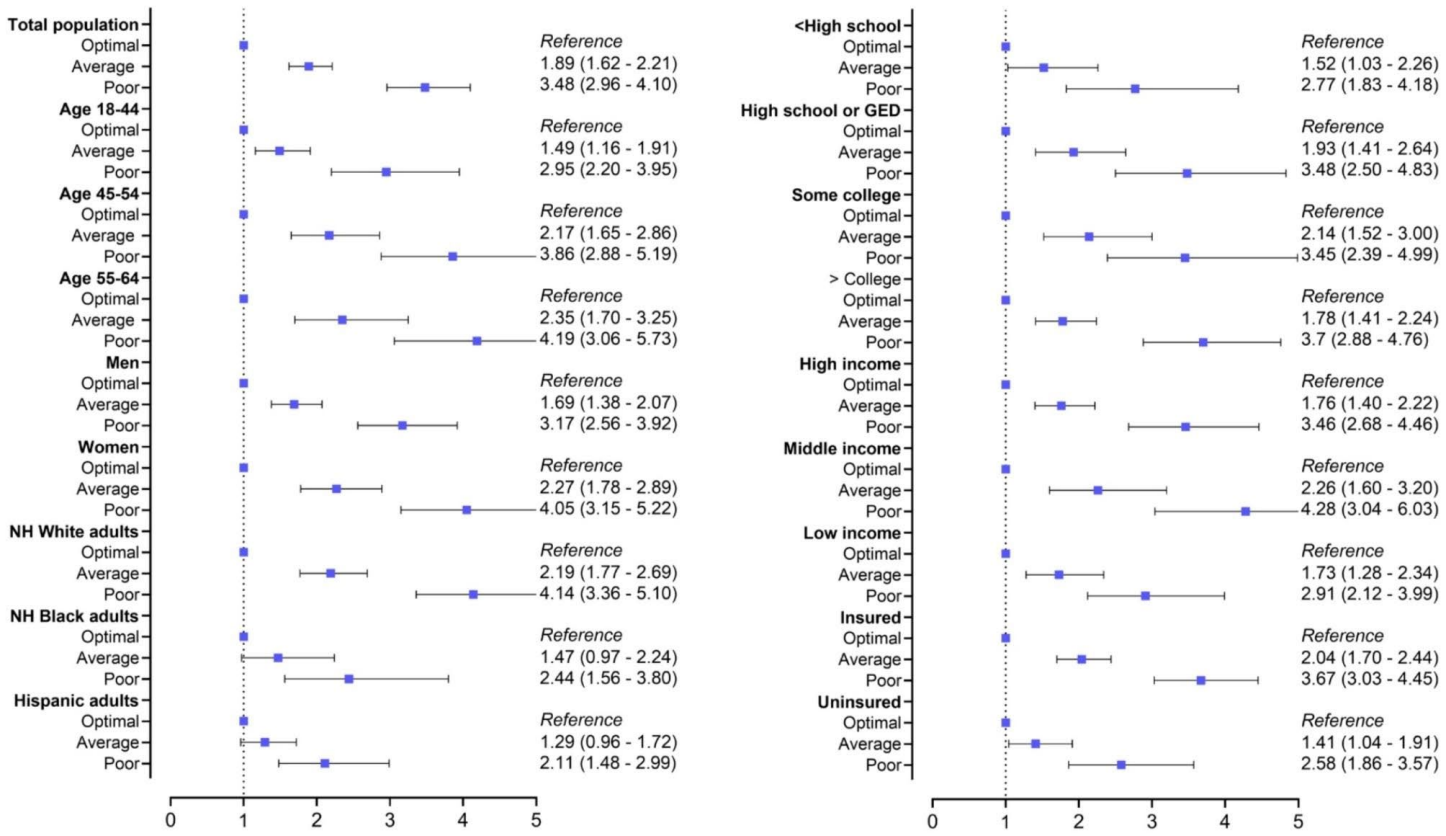
All-Cause Mortality Hazard Ratios by Sociodemographic subgroups, from National Interview Health Survey 2006–2014

Results from Cox proportional hazards regression analysis are presented in Table 3. We noted a stepwise increase in adjusted (for age, sex, race/ethnicity and education) HR for all-cause mortality across CRF profiles: average vs. optimal CRF profiles (aHR 1.89; 95% CI 1.62–2.21) and poor vs. optimal CRF profiles (aHR 3.48; 95% CI 2.96–4.10). This pattern of incremental all-cause mortality risk with worsening CRF profile was consistently observed for both young and middle aged adults, and across racial/ethnic (NHW, NHB, Hispanic and sex (male, female)) subgroups. All-cause mortality hazard ratios by sociodemographic subgroups and CRF profile (reference: optimal CRF) are depicted in Fig. 3.

**Table 3A:** Risk of All-cause and CVD mortality by Sociodemographic Subgroups, NHIS 2006–2014

		All-Cause Mortality		Cardiovascular Disease Mortality	
		Hazard Ratio (95% CI)		Hazard Ratio (95% CI)	
	CRF Profile	Model 1*	Model 2**	Model 1*	Model 2**
	Optimal	Reference	Reference	Reference	Reference
Total Population	Average	2.57 (2.26–2.92)	1.89 (1.62–2.21)	5.16 (4.08–6.53)	2.86 (2.09–3.92)
	Poor	7.68 (6.71–8.80)	3.48 (2.96–4.10)	13.43 (10.57–17.08)	4.76 (3.44–6.60)
Age: 18–44 y	Average	1.97 (1.62–2.38)	1.49 (1.16–1.91)	6.31 (2.56–15.59)	3.99 (1.08–14.74)
	Poor	4.84 (3.81–6.13)	2.95 (2.20–3.95)	24.58 (9.51–63.50)	10.81 (2.67–43.69)
45–54 y	Average	2.56 (2.01–3.25)	2.17 (1.65–2.86)	3.46 (1.84–6.51)	2.96 (1.26–6.96)
	Poor	5.43 (4.21–7.01)	3.86 (2.88–5.19)	7.58 (3.96–14.50)	4.95 (2.12–11.54)
55–64 y	Average	2.72 (2.07–3.58)	2.35 (1.70–3.25)	2.88 (1.50–5.54)	2.64 (1.14–6.08)
	Poor	5.89 (4.54–7.65)	4.19 (3.06–5.73)	6.74 (3.58–12.70)	5.74 (2.54–12.93)
Sex: Men	Average	2.51 (2.12–2.97)	1.69 (1.38–2.07)	4.09 (3.02–5.53)	2.63 (1.78–3.89)
	Poor	7.16 (6.01–8.53)	3.17 (2.56–3.92)	11.51 (8.47–15.63)	4.56 (3.04–6.83)
Women	Average	2.69 (2.19–3.29)	2.27 (1.78–2.89)	7.41 (5.06–10.84)	3.29 (2.01–5.37)
	Poor	8.61 (7.00–10.60)	4.05 (3.15–5.22)	17.83 (12.10–26.27)	5.23 (3.18–8.60)
NH White adults	Average	3.19 (2.64–3.85)	2.19 (1.77–2.69)	5.9 (4.51–7.72)	3.1 (2.21–4.34)
	Poor	10.15 (8.42–12.24)	4.14 (3.36–5.10)	13.85 (10.50–18.28)	5.18 (3.65–7.34)
NH Black adults	Average	2.62 (1.80–3.81)	1.47 (0.97–2.24)	4.54 (2.21–9.33)	1.57 (0.70–3.54)
	Poor	7.93 (5.45–11.54)	2.44 (1.56–3.80)	14.6 (7.25–29.39)	2.34 (1.04–5.25)
Hispanic adults	Average	1.64 (1.27–2.11)	1.29 (0.96–1.72)	3.85 (1.80–8.27)	2.01 (0.74–5.46)
	Poor	3.62 (2.66–4.93)	2.11 (1.48–2.99)	14.9 (6.87–32.32)	4.04 (1.42–11.50)
<High school	Average	2 (1.46–2.73)	1.52 (1.03–2.26)	5.16 (2.91–9.16)	2.67 (1.36–5.28)
	Poor	5.71 (4.15–7.86)	2.77 (1.83–4.18)	12.34 (6.89–22.07)	4.09 (2.05–8.17)
High school or GED	Average	2.18 (1.65–2.88)	1.93 (1.41–2.64)	3.76 (2.26–6.27)	2.77 (1.47–5.20)
	Poor	5.84 (4.42–7.72)	3.48 (2.50–4.83)	7.43 (4.48–12.33)	4.32 (2.31–8.08)
Some college	Average	2.95 (2.22–3.91)	2.14 (1.52–3.00)	4.29 (2.57–7.14)	2.33 (1.26–4.32)
	Poor	8.95 (6.61–12.12)	3.45 (2.39–4.99)	13.81 (8.34–22.87)	4.45 (2.46–8.08)
> College	Average	2.25 (1.86–2.71)	1.78 (1.41–2.24)	4.69 (3.19–6.91)	2.93 (1.83–4.70)
	Poor	6.65 (5.37–8.24)	3.7 (2.88–4.76)	12.38 (8.19–18.73)	5.31 (3.19–8.83)
High income	Average	2.18 (1.77–2.68)	1.76 (1.40–2.22)	6.22 (3.67–10.56)	3.09 (1.77–5.41)
	Poor	5.55 (4.42–6.99)	3.46 (2.68–4.46)	15.12 (8.73–26.19)	5.11 (2.90–9.02)
Middle income	Average	3.01 (2.21–4.10)	2.26 (1.60–3.20)	7.03 (4.30–11.50)	3.79 (2.25–6.37)
	Poor	8.57 (6.33–11.61)	4.28 (3.04–6.03)	17.03 (10.30–28.18)	5.91 (3.46–10.09)
Low income	Average	2.39 (1.83–3.12)	1.73 (1.28–2.34)	3.88 (2.54–5.95)	1.87 (1.18–2.95)
	Poor	7.3 (5.56–9.58)	2.91 (2.12–3.99)	11.33 (7.33–17.50)	3.19 (1.99–5.11)
Insured	Average	2.91 (2.48–3.40)	2.04 (1.70–2.44)	5.85 (4.53–7.54)	2.89 (2.09–3.98)
	Poor	9.24 (7.86–10.86)	3.67 (3.03–4.45)	14.57 (11.23–18.92)	4.77 (3.41–6.67)
Uninsured	Average	1.48 (1.15–1.90)	1.41 (1.04–1.91)	2.39 (1.13–5.05)	2.67 (0.91–7.80)
	Poor	3.63 (2.82–4.65)	2.58 (1.86–3.57)	6.81 (3.17–14.61)	5.52 (1.81–16.86)

Abbreviations: HR, Hazard Ratios; CI, confidence interval

* Unadjusted

** Adjusted for age, sex, race/ethnicity, education, income, and insurance status

Note: Optimal CRF Profile = 0 CV risk factors; Average = 1–2 risk factors; Poor ≥ 3 risk factors

**Table 3B:** Sensitivity Analysis Sensitivity analysis:

		All-Cause Mortality		Cardiovascular Disease Mortality	
		Hazard Ratio (95% CI)		Hazard Ratio (95% CI)	
	CRF Profile	Model A*	Model B**	Model A*	Model B**
	Optimal	Reference	Reference	Reference	Reference
Total Population	Average	2.15 (1.81–2.57)	1.21 (0.80–1.83)	2.56 (1.58–4.14)	2.53 (0.69–9.25)
	Poor	3.91 (3.24–4.72)	1.64 (1.05–2.55)	6.51 (3.97–10.68)	2.08 (0.56–7.751)40

*Adjusted for age, sex, race/ethnicity, education, income, insurance status, region, delayed/foregone care due to cost, food insecurity

**Adjusted for age, sex, race/ethnicity, education, income, insurance status, region, delayed/foregone care due to cost, food insecurity, ASCVD, cancer, sleep trouble

#### Association between cardiovascular risk factor (CRF) profile and CVD mortality

**Fig. 4:**
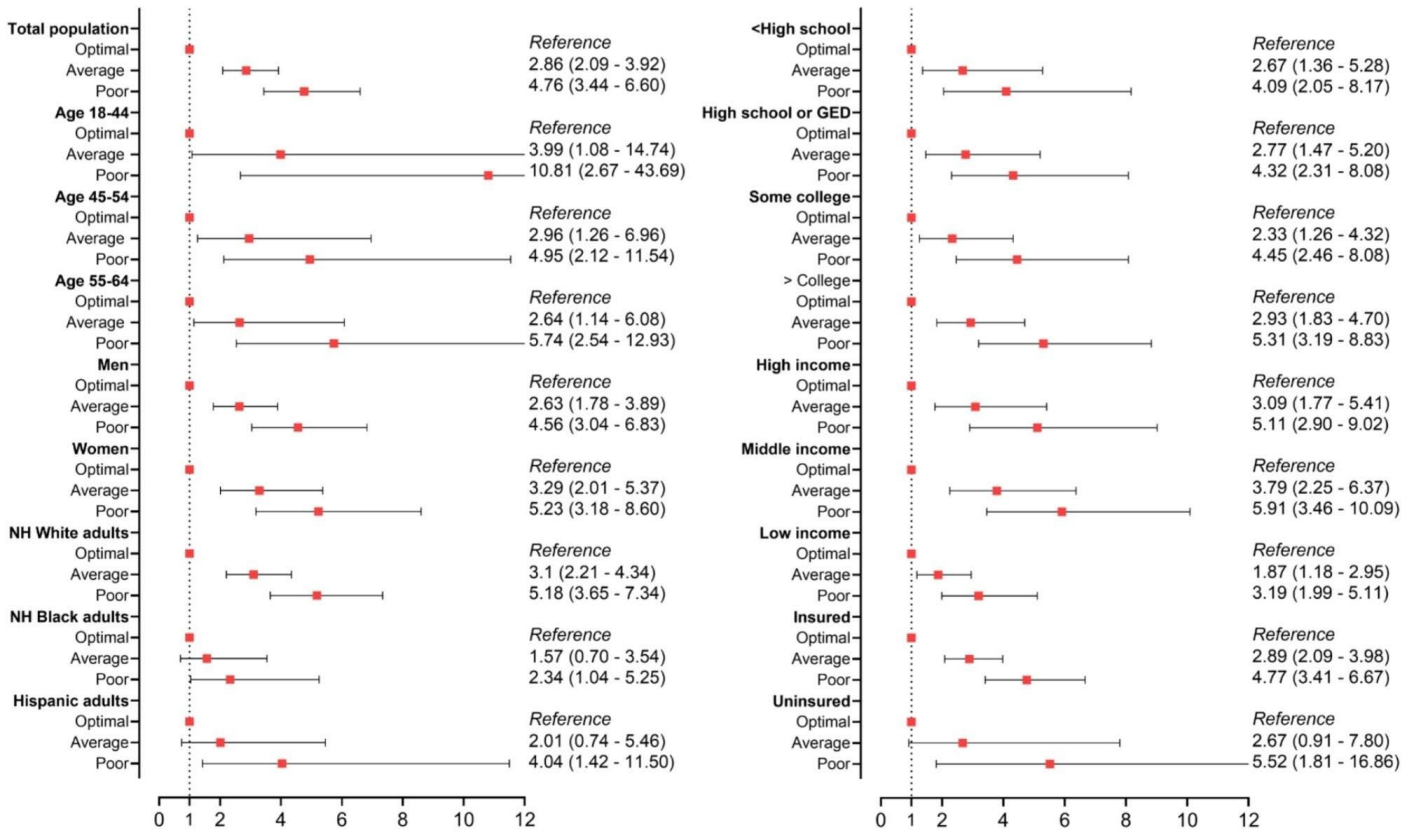
Cardiovascular Mortality Hazard Ratios by Sociodemographic Subgroups, from National Interview Health Survey, 2006–2014

There was a stepwise increase in risk of for CVD mortality across worsening CRF profile. Poor and average CRF profile was associated with nearly three to five fold increased risk of CVD mortality in fully adjusted models: adjusted HR, average vs. optimal CRF profiles (aHR 2.86, 95% CI 2.09–3.92) and poor vs. optimal CRF profiles (aHR 4.76, 95% CI 3.44–6.60) (Table 3). Similar trends were observed for sociodemographic subgroups. The observed CRF – mortality association was relatively stronger for the elderly, females, and NH White subgroups, relative to their counterparts; however, the risk of mortality across each sociodemographic subgroup was consistently and significantly higher for individuals with poor CRF profile relative to those with optimum profile. CVD mortality HRs by sociodemographic and CRF profiles (reference: optimal) are depicted in Fig. 4.

In sensitivity analyses, we found that the association between CRF and premature mortality attenuated after additional adjustment for SDoH and comorbidities. However, overall, we found a similar pattern of higher premature mortality risk with increasing CRF burden (Table 3B).

## Discussion

In this nationally representative study of non-elderly US adults (< 65 years age), we found that 16.2% of all participants had poor CRF profile. We also found that NH Black middle-aged adults (45–64), and those with lower levels of education and income were more likely to experience poor CRF profile compared with other sociodemographic subgroups. We observed a stepwise increase in the risk of all-cause and CVD mortality across worsening CRF risk profile categories, such that participants with poor CRF profiles had a 2–3 fold higher risk of all-cause and 4–5 fold higher risk of CVD mortality, compared to those with optimal CRF profile; these patterns were consistently observed among age, sex and racial/ethnic subgroups.

Our findings corroborate prior evidence which suggests that conventional cardiac risk factors such as hypertension, diabetes, hyperlipidemia, smoking, lack of physical activity, and obesity are independently associated with, and incrementally increase the risk of CVD and CVD mortality [18–21]. Despite the existing evidence of the role of individual risk factors in determining CVD and/or mortality risk, prior studies have not assessed the risk of all-cause and CVD mortality using a comprehensive, composite measure of CRF profile.

Our findings have important implications for improving CVD outcomes on a population level. For instance, Ford et al. highlighted that controlling traditional cardiac risk factors can lead to a nearly 50% reduction in deaths from coronary heart disease [22]. Our study revealed that individuals carrying a poor CRF profile (≥ 3 risk factors) had higher CVD and all-cause mortality compared to those with an average or optimal CRF profile. Additionally, our study provides important insights into the impact of worsening CRF profile on both CVD and all-cause mortality in the understudied non-elderly (< 65 years age) population.

In a study involving 6,229 healthy participants aged 44–84 enrolled in the Multi-Ethnic Study of Atherosclerosis (MESA), participants with higher number of unfavorable CRF (smoking, obesity, diet and physical activity) had an incremental burden of subclinical atherosclerosis as measured by coronary artery calcium progression as well as an incremental burden of all-cause mortality at a median follow-up of 8 years [23]. These findings point towards the importance of identifying and quantifying CRF in order to guide patient-specific risk factor modification strategies, especially in the economically productive non-elderly adults who may not be aware of their cardiovascular risk profile until the onset of clinically significant CVD [24]. This is especially important given the rising burden of cardiovascular risk factors and the fact that ~ 50% of US adults had at least one uncontrolled cardiovascular risk factor between 1999 and 2010 [3]. However, efforts to target individual modifiable risk factors to curb the rise in CVD rates have been insufficient, [25] and interventions such as the Million Hearts Program are expected to fall short of their long-term targets of reducing the incidence of first-time heart attacks, strokes, or transient ischemic attacks [26].

The American Heart Association proposed that Americans follow “Life’s Essential 8 (LE8)” for primary prevention of overall cardiovascular disease. Life’s Essential 8 measures overall cardiovascular health on a point scale (0-100) based on cardiovascular disease risk factors or behaviors, including: diet, physical activity, nicotine exposure, sleep health, body mass index, blood lipids, blood glucose, and blood pressure. Since then, studies have correlated the association of ideal LE8 metrics with decreased risk of CVD [27]. On the same account, our findings of using a CRF profile score validate the discernibility of a composite metric in evaluating the CVD risk of a large, representative real world population. Our results highlight that approximately 16% of non-elderly adults in the US have poor CRF profile; it is even more concerning to note that these participants have a 2–3 fold higher all-cause and 4–5 fold higher CVD mortality risk. These findings points toward the need for urgent population level interventions to curb persistently high premature mortality rates in the US.

The effect of adverse social determinants of health (SDoH) such as housing, education, income, race and ethnicity on the prevalence of these risk factors merits equal attention [28]. Our study showed that individuals with a high school degree or lower and with a low-middle family income had higher age-adjusted all-cause and CVD mortality rates across all CRF profiles compared to those with a college degree or higher and high family income. Additionally, we observed that participants with a poor CRF profile were ~ 2 times likely to be NH Black adults compared with participants with an optimal CRF profile, and that the former were half as likely to have a college level education or above. We also observed differences in all-cause and CVD mortality by region with the highest rates for all-cause mortality noted in the South (poor CRF profile) and CVD-mortality in the Midwest (poor CRF profile). The interplay between race/ethnicity and upstream SDoH factors such as place of living/region, educational attainment and income may impact an individual’s ability to seek primary preventive care, buy healthy food, live in neighborhoods with sufficient recreation and physical activity avenues, and adhere to guideline directed medical therapy [29]. Additional studies are needed to fully understand the mediating and moderating role of individual SDoH, as well as the intersectionality between race and SDoH, for risk of mortality.

In this study, we presented AAMR across separate CRF profile categories, which are composed of individual cardiovascular risk factors that are established predictors of mortality and drivers of racial/ethnic disparities in mortality. Given that our CRF index is a composite of such established risk factors, it is possible that “accounting for” these risk factors attenuated and/or reversed the racial/ethnic disparity in mortality rates, such as relatively higher AAMR for non-Hispanic White adults compared to non-Hispanic Black adults at “poor CRF” category. However, these patterns should be interpreted with caution, given that AAMR are descriptive statistics and with the observed overlap in confidence intervals especially for NHB vs NHW subgroups, the statistical significance of such variation cannot be firmly established.” Additional study is needed to further explore these patterns.

Healthcare systems and providers alike should assess a patient’s cardiovascular disease risk holistically—taking into account both their composite risk factors and upstream socioeconomic factors such as race/ethnicity, education, income, and insurance status—in order to better understand and identify higher risk patients. Cardiovascular care has become increasingly costly with studies highlighting that patients with established ASCVD have high financial burdens [30]. Beyond cardiovascular disease, many Americans also suffer from other co-morbidities and our study highlighted that an increasing number of co-morbidities led to an increase in both all-cause and CVD mortality across all CRF profiles. Composite CRF indices such as the one presented in this study highlight the need to consider the composite effects of clinical and behavioral risk factors for a holistic assessment of mortality/premature mortality risk. Our approach may inform the development and potential application of similar tools – also including additional behavioral risk factors such as alcohol use and dietary patterns – to assess patients’ risk in real-world settings. Given that most CRF information is readily available in electronic health record databases, such a risk score may help stratify patients’ cumulative risk of cardiovascular disease, which in turn may inform much needed efforts for primary and secondary prevention of CVD, including identification, screening, and early treatment of clinically vulnerable patients to prevent the risk of CVD and associated outcomes including mortality downstream.

### Study limitations

Our findings should be perceived in light of certain limitations. First, this was a cross-sectional analysis, hence, we could not infer causality. Future evidence from longitudinal studies is needed to account for potential temporal variation in CRF burden. Second, NHIS data is self-reported and inherently susceptible to recall-bias. However, NHIS is subject to multiple quality checks to ensure accuracy and reliability of the reported data. Further, the data is routinely used in epidemiologic investigation informing research and policy nationally [24]. Since the NHIS only contains data on the non-institutionalized population, our findings cannot be generalized to the institutionalized population. The latter have been shown to carry a higher CVD risk than community dwelling participants. Additionally, our study created a comprehensive CRF profile measure that included not only established clinical risk factors such as Diabetes, Hypertension, High Cholesterol and Obesity but also included behavioral determinants of cardiovascular health including Insufficient Physical Activity and Smoking. However, given limitations of NHIS data used herein, we could not include other important risk factors such as diet and alcohol intake. These risk factors merit greater study in additional databases in the future. Finally, while we adjusted for known sociodemographic and clinical variables, the possibility of residual confounding cannot be ruled out in any observational study. Despite these limitations, the NHIS data continue to serve as a reliable repository for population level estimates of leading health indices in the US.

## Conclusions

Although it is well documented in the literature that individual cardiac risk factors (hypertension, diabetes, hyperlipidemia, smoking, sedentary lifestyle, and obesity) are independently associated with CVD, our data shows that poor composite CRF profile is associated with a 3-fold higher risk of all-cause and 5-fold higher risk of CVD mortality. The effect of worsening CRF profile on mortality was seen consistently across key sociodemographic subgroups. Socially disadvantaged subgroups, including those with low income, lower education and NHB adults were more likely to experience poor CRF profile, compared to their counterparts. Our findings highlight the usefulness of a comprehensive cardiovascular risk index in identifying non-elderly individuals with high risk of mortality, based on their CRF profile. Our approach and results may inform future CVD prevention efforts to target clinically susceptible individuals, and reduce their risk of premature mortality.

## Data Availability

All data used in this study are available below: 1. Centers for Disease Control and Prevention. (2022, September 7). NHIS - National Health Interview Survey. Centers for Disease Control and Prevention. Retrieved September 12, 2022, from https://www.cdc.gov/nchs/nhis/index.htm. 2. Centers for Disease Control and Prevention. (2022, January 10). Data Access - National Death index. Centers for Disease Control and Prevention. Retrieved October 26, 2022, from https://www.cdc.gov/nchs/ndi/index.htm.
